# Optofluidic variable optical path modulator

**DOI:** 10.1038/s41598-019-43599-4

**Published:** 2019-05-08

**Authors:** Qiong-Hua Wang, Liang Xiao, Chao Liu, Lei Li

**Affiliations:** 1 School of Instrumentation and Optoelectronic Engineering, Beihang University, Beijing, 100191 China; 2 School of Electronics and Information Engineering, Sichuan University, Chengdu, 610065 China

**Keywords:** Optofluidics, Adaptive optics

## Abstract

The optofluidic devices including optofluidic lens, optical switch and liquid prism have found widespread applications in imaging, optical communication and lighting. Here, we report a novel optofluidic device called optofluidic variable optical path modulator. Our proposed modulator consists of two main chambers. The two chambers are connected by two tubes to form a closed-loop fluidic system. Two immiscible liquids are filled into the two chambers and form two L-L interfaces. A transparent sheet is placed between one L-L interface to get flat interface. When a voltage is applied on the device, the flat interface can move up and down. Thus, variable optical path can be obtained by applying a voltage. To prove the concept, we fabricate an optofluidic device whose largest movable distance of L-L interface is ~7.5 mm and the optical path length change is ~1.15 mm. The proposed optofluidic device has potential applications in imaging, adaptive optics, optical detection and so on.

## Introduction

A variable optical path modulator is a crucial part for many optical systems. For example, adaptive optics system needs variable optical path to compensate the aberrations which result from atmospheric disturbance^1^. Spatial light modulator (SLM) needs variable optical path to generate the variable phase^2^. Imaging systems also need variable optical path to compensate the back focal length^3^. To conclude, variable optical path is an essential technology to realize precise optical adjustment. Unfortunately, in these systems, variable optical path is generated either by mechanically shifting the position of the optical elements or by a variable refractive index material (e. g. Liquid crystal). Therefore, they are either bulky, or with limited optical path tuning range. For example, the SLM usually has only a tuning range of several wavelengths. Thus, it is still urgent to develop new variable optical path modulator.

Fortunately, optofluidics provides a new way to develop tunable optical devices. Optofluidics relies on the use of controllably movable and deformable liquid interfaces to realize particular optical functionality. Due to characteristic of the liquid and shape tunable ability, optofluidic devices^4–14^ have the merits of polarization-independence, broadband, high transmittance and fast response. Therefore, many optofluidic devices are designed to replace the conventional solid devices such as optofluidic lenses^4–10^, optical switches^11,12^ and liquid prisms^13,14^. Due to deformable L-L (liquid - liquid) interfaces, the optofluidic devices can achieve many optical functionalities: variable focal length, variable aperture and beam steering. And, it is possible to get a variable optical path based on optofluidics. For example, a deformable transparent optofluidic wavefront modulator is proposed to correct the wavefront error by controlling the polymer membrane^15^. An optofluidic light modulator in lab-on-a-chip is proposed^16^. It can modulate the incident light with different shapes for micromachining. An optofluidic modulator based on peristaltic nematogen microflows is proposed^17^. Due to peristaltic nematogen microflows, the optofluidic modulator realize a fast response time. Red blood cells are used as adaptive optofluidic microlenses for endoscopic vision^18^. These devices^15–18^ based on optofluidics can modulate the light by varying the optical path and changing the shape of the beam, which is very suitable for micro-imaging and micro-machining technology. However, the tuning range of optical path is small. Thus, the optofluidic modulating devices with large tuning range are also needed.

Here, we show a new device developed with the techniques of the optofluidics, optofluidic variable optical path modulator. Our proposed modulator consists of two main chambers. The two chambers are connected by two tubes to form a closed-loop fluidic system. A transparent sheet is placed between one L-L interface to get flat interface. When a voltage is applied on the device, the flat interface can move up and down. Thus, variable optical path can be obtained by applying a voltage. The largest movable distance of L-L interface is ~7.5 mm, which generates the optical path length change of ~1.15 mm. Comparing with the conventional optofluidic modulating devices, the proposed device can get a large optical path tuning range with relatively compact structure, and easy operation.

## Schematic and Principle

**Figure 1:**
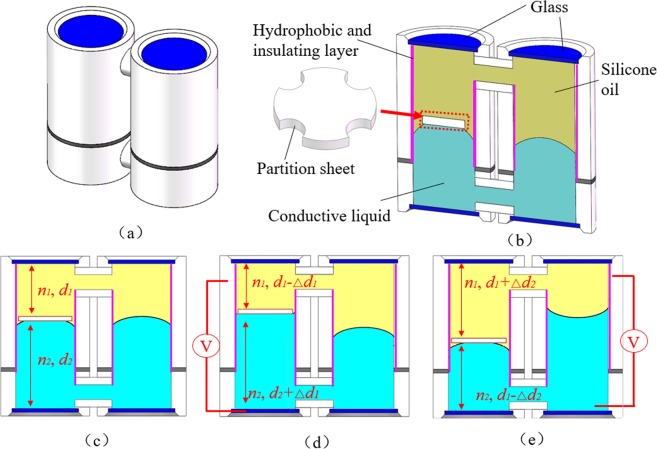
Schematic cross-sectional structure and operating mechanism of our optofluidic modulator: (**a**) Whole view. (**b**) Sectional view. A transparent sheet is placed between one L-L interface to form a flat interface. (**c**) Initial state. (**d**) Moving up state. When the external voltage *U*_1_ is applied on the device, the L-L interface moves upwards. (**e**) Moving down state. When the external voltage *U*_2_ is applied on the device, the L-L interface moves downwards.

The cross-sectional cell structure and the operating mechanism of our proposed modulator are depicted in Fig. 1.

The proposed modulator consists of two main chambers, as shown in Fig. 1(a,b). The two chambers are connected by two tubes to form a closed-loop fluidic system. The inner wall of the chamber is coated with a hydrophobic and insulating layer. The lower part of the two chambers is filled with a conductive liquid, and the rest space is filled with silicone oil. Different from the conventional optofluidic devices, a transparent sheet is placed between one L-L interface. Therefore, a flat interface is formed in the left chamber. The left chamber is the effective part to modulate optical path, and the right chamber is used to form the closed-loop fluidic system. In the initial state, the whole optical path consists of two parts: oil phase and water phase, as shown in Fig. 1(c). The whole optical path length is *n*_1_*d*_1_ + *n*_2_*d*_2_. When a voltage is applied on the left electrode, the L-L interface deforms from convex shape to concave shape, which generates fluidic flow upwards due to electrowetting effect. Thus, the flat interface moves up in the left chamber, as shown in Fig. 1(d). Due to different refractive index of silicone oil and conductive liquid, the whole optical path is changed. If the shifted distance is *Δd*_1_, the whole optical path length becomes *n*_1_*d*_1_ + *n*_2_*d*_2_ + (*n*_2_ − *n*_1_)*Δd*_1_. When a voltage is applied on the right electrode, in the same way, the conductive liquid flows upwards due to electrowetting effect in the right chamber, which pushes the silicone oil in the left chamber down. Thus, the flat interface descends in the left chamber, as shown in Fig. 1(e). In this case, if the shifted distance is *Δd*_2_, the whole optical path length becomes *n*_1_*d*_1_ + *n*_2_*d*_2_ + (*n*_1_ − *n*_2_)*Δd*_2_. In this way, the whole optical path in the left chamber can be tuned by a voltage. Thus, we get a new optofluidic device: optofluidic variable optical path modulator.

For our optofluidic modulator, the position of the L-L interface is changed due to electrowetting effect^4^. According to Young–Lippmann equation, the relationship of the contact angle *θ* and the applied voltage *U* can be described as follows:

1 $$\cos \,\theta =\,\cos \,{\theta }_{{\rm{0}}}+\frac{\varepsilon }{2{\gamma }_{12}d}{U}^{2},$$

where ε is the dielectric constant of the insulating layer, *d* is the thickness of the insulating layer, *γ*_12_ is the interfacial tensions of silicon oil/conductive liquid. *θ*_0_ is initial contact angle.

## Results and Discussions

### Operating process

**Figure 2:**
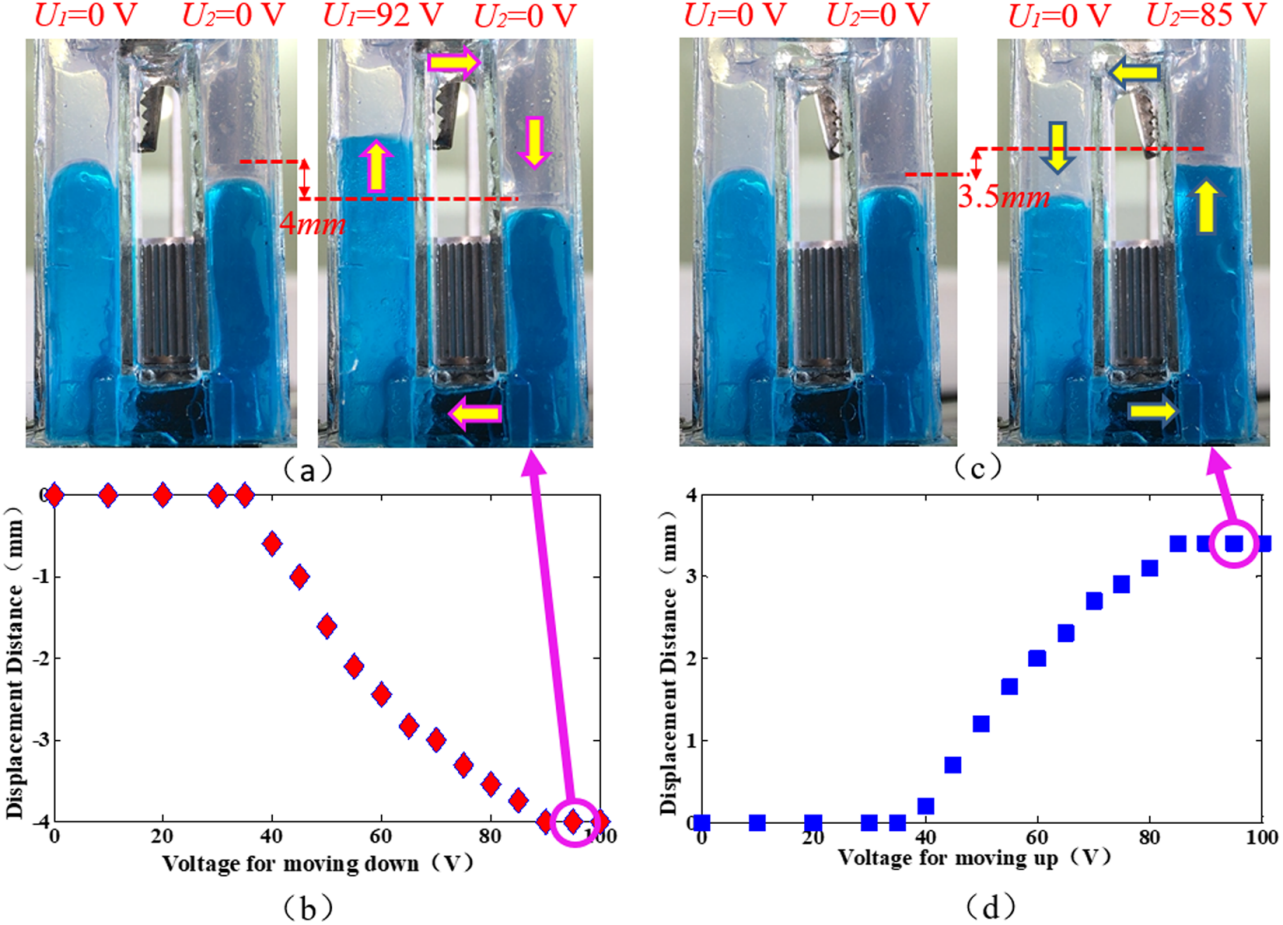
Performance of moving behavior of the proposed optofluidic modulator. (**a**) Clockwise liquid flows. (**b**) Moving distance (downwards) with different voltages. (**c**) Counter-clockwise liquid flows. (**d**) Moving distance (upwards) with different voltages.

To visually demonstrate the moving behavior of the L-L interface, we first fabricate the device using transparent materials. We note that the transparent cell is not the final device, and it is only used for demonstrating the moving behavior. To fabricate the two chambers, four rectangular ITO (indium tin oxide) glass sheets (8 mm × 50 mm) coated with 3-μm Parylene-C+ Teflon are assembled to form a cubic chamber. Two cubic chambers are connected by two tubes. The material of the transparent sheet is PMMA (polymethylmethacrylate). We inject conductive liquid into the two chambers. The conductive liquid is NaCl solution, and its density is ~1.09 g/cm^3^ (refractive index *n*_1_ = 1.35). To observe the behavior of the L-L interface clearly, we dye the conductive liquid with blue pigment. Then we fill the rest space with silicone oil. The density of the silicon oil is ~1.09 g/cm^3^ (refractive index *n*_2_ = 1.50). A CCD camera is placed on the lateral side of the device. It is used to record the position change of the L-L interface during actuation. Figure 2 shows the recorded result when different voltages are applied on the device. We note that the flat interface is in the right chamber. In the experiment, we first apply a voltage *U*_1_ on the left chamber. The driving voltage of the device is ~36 V. Below 36 V, the flat interface keeps still. When a voltage larger than 36 V is applied, for example 40 V, the L-L interface in the left chamber moves up, which generates clockwise liquid flows. Thus, the flat interface in the right chamber descends. When we further increase the driving voltage, the displacement of the flat interface is larger. When the voltage *U* > 92 V, the shifting distance remains nearly the same, because the contact angle of the conductive liquid in the left chamber is saturated, as shown in Fig. 2(a,b). The largest displacement is measured to be ~4.0 mm. The moving speed of the L-L interface for descending is measured to be ~7.14 mm/s. A dynamic response video of the moving down behavior at *U*_1_ = 92 V is also included (Media 1). When a voltage *U*_2_ is applied on the right chamber, the electrowetting effect happens in the right chamber which generates counter-clockwise liquid flows. Therefore, the flat interface moves up, as shown in Fig. 2(c,d). When the voltage increases to ~85 V, the contact angle is saturated. The largest displacement is measured to be ~3.5 mm. The moving speed of the L-L interface is measured to be ~7.29 mm/s. A dynamic response video of the moving up behavior at *U*_2_ = 85 V is also included (Media 2). From the experiment, we can conclude that the proposed optofluidic modulator can move the flat interface up and down, which results in tunable optical path.

To test the repeatability of the device, we apply DC and AC voltages on the device for 300 times and measure the displacement of the L-L interface, respectively. The results are shown in Table 1. We can find that the displacement of the L-L interface slightly changes by ~0.2 mm (downwards) and ~0.1 mm (upwards) for the DC voltage. However, for the AC voltage, the displacement of the L-L interface remains the same. The main reason is that the electric charge accumulates in the Parylene-C+ Teflon layer after applying voltage for several times, which prevents the contact angle from changing. However, for the AC voltage, such electric charge accumulation phenomenon does not exist. Thus, the device is stable.

**Table 1:** The displacement of the L-L interface for AC and DC voltages.

Times	1	50	100	150	200	250	300
Downwards (92 V DC)	~4.0 mm	~4.0 mm	~3.9 mm	~3.9 mm	~3.9 mm	~3.9 mm	~3.8 mm
Downwards (92 V AC)	~4.0 mm	~4.0 mm	~4.0 mm	~4.0 mm	~4.0 mm	~4.0 mm	~4.0 mm
Upwards (85 V DC)	~3.5 mm	~3.5 mm	~3.4 mm	~3.4 mm	~3.4 mm	~3.4 mm	~3.4 mm
Upwards (85 V AC)	~3.5 mm	~3.5 mm	~3.5 mm	~3.5 mm	~3.5 mm	~3.5 mm	~3.5 mm

We note that the left and right chamber modulations have different saturation voltage and displacement. The main reason is that in the right chamber a transparent sheet is placed between the L-L interface to get a flat interface to avoid aberrations. The sheet results in different initial contact angles *θ*_0_ in the two chambers. According to Eq. (1), the parameters *θ*_0_ for the two chambers are different, thus the saturation voltages *U* are different, which results in different displacement of L-L interface.

### Fabrication and assembly

**Figure 3:**
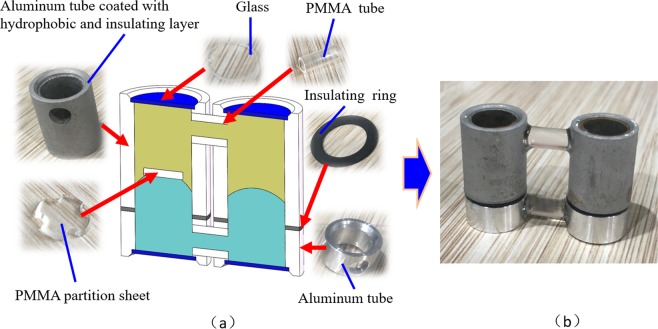
Fabricated prototype of the optofluidic modulator. (**a**) All the elements of the device. (**b**) Assembled prototype.

To evaluate the performance more accurately, we fabricate a prototype of the optofluidic variable optical path modulator by precision machining. The prototype consists of 6 kinds of elements, as shown in Fig. 3(a). The chamber is the main part of the device. The upper part of the chamber is an aluminum tube coated with 3-μm Parylene-C+ Teflon layer. The lower part is an aluminum tube without coating, which serves as an electrode. Between the two tubes, there is an insulating ring to separate the two parts. The material of the top and bottom window glass is BK7 in glass data of SHOTT^19^. The diameter of the window glass is 9 mm. The four elements are stuck together to form a chamber. The size of the chamber is 12 mm (diameter) × 21.5 mm (height). The transparent sheet is fabricated by engraving machine and the material is PMMA. Two PMMA tubes are used to connect the two chambers. The diameter of the tube is 3.5 mm. The conductive liquid is NaCl solution, and its density is ~1.09 g/cm^3^ (refractive index *n*_1_ = 1.35). The other liquid is the silicon oil with the density of ~1.09 g/cm^3^ and refractive index is 1.50. The whole device is shown in Fig. 3(b).

### Measurement of the optical path length

**Figure 4:**
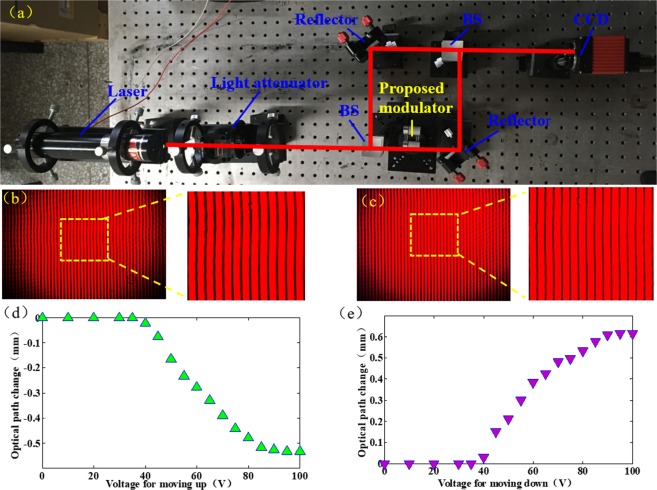
Measurement of the optical path change. (**a**) Setup of the measurement system. (**b**) Fringe pattern in the initial state. (**c**) Fringe pattern at 60 V. (**d**) Optical path change at different voltages for moving up state. (**e**) Optical path change at different voltages for moving down state.

To measure to the optical path accurately, we set up an optical interference system. The experimental setup is shown in Fig. 4(a). A laser beam splits into two beams by a BS (beam splitter). One beam passes through the proposed modulator, and the other beam is reflected by the reflector. The two beams finally merge at the last BS and interfere with each other. A fringe pattern is generated by the two beams, which contains the information of optical path. A CCD camera is the used to record the fringe pattern. In the initial state, we obtain the fringe pattern, as shown in Fig. 4(b). When we apply a voltage ~60 V on chamber with flat interface, the recorded fringe pattern is changed, which indicates that the optical path length varies, as shown in Fig. 4(c). The two fringe patterns are analyzed by a Fourier-transform method^20,21^ to calculate the accurate optical path difference. In the experiment, we apply different voltages on the device to make the flat interface move up and down and record the fringe patterns. After calculating, the results are shown in Fig. 4(d,e). When we gradually increase the voltage on the chamber with the flat interface, the flat interface moves up. Thus, in the tube, the path length of oil phase is shortened, but the path length of water phase lengthens. Because the refractive index of conductive liquid is larger than that of silicone oil, the total optical path length is shorten. The largest optical path length change is measured to be ~0.53 mm. When we gradually increase the voltage on the other chamber, the flat interface descends, which results in increase of the optical path length. The largest optical path length change is measured to be ~0.62 mm. From the experiment, we can conclude that the proposed optofluidic modulator can get variable optical path length. The total largest optical path length change is ~1.15 mm.

### Application in imaging system

**Figure 5:**
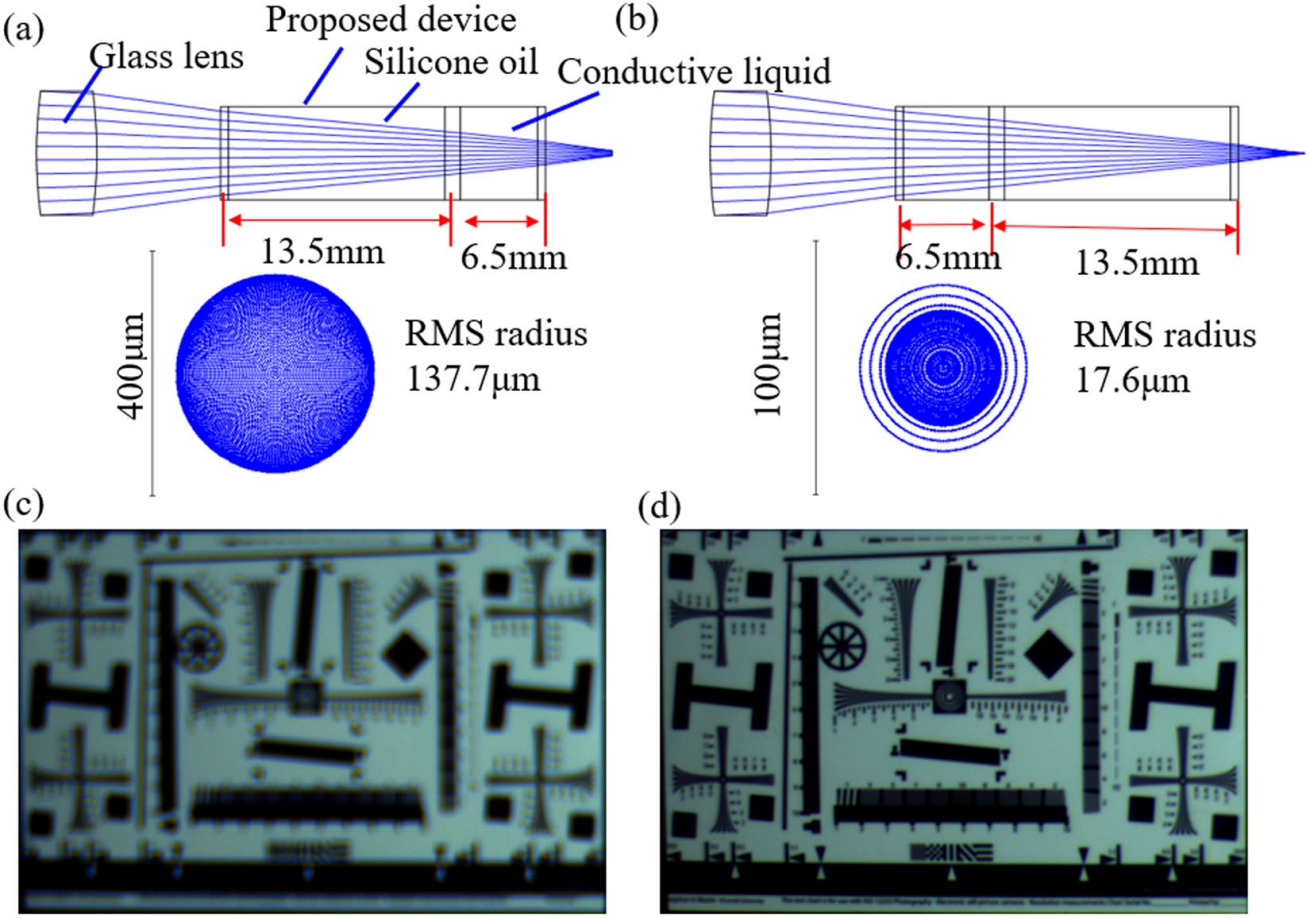
Application in imaging system. (**a**) Zemax model in the initial state. (**b**) Zemax model in the focal state. (**c**) Image quality when a voltage (~90 V) is applied on the chamber without the flat interface. (**d**) Image quality when a voltage (~82 V) is applied on the chamber with the flat interface.

The proposed modulator can be used in an imaging system to compensate the back focal length. We set up an imaging system, which consists of a glass lens and the proposed modulator. The glass lens is a convex lens. The material is K9. The focal length is 30 mm. The diameter of the lens is 8 mm. In the system, the distance between the glass lens and the proposed modulator is 8 mm. The back focal length is 5.5 mm. A CCD camera is used as an image plane. The resolution of the camera is 2592 × 1944. We model the system in Zemax^22^. In the initial state, the image is out of focus. Thus, the ray traced spot is blurring, as shown in Fig. 5(a). The RMS radius of the spot is ~137.7 μm. When the flat interface is moved forward ~7 mm, we can get a much clearer image, as shown in Fig. 5(b). The RMS radius of the spot reduces to ~17.6 μm. From the simulation, we can conclude that the proposed modulator can compensate the back focal length effectively. We also do an imaging experiment using the system. In initial state, we apply a voltage (~90 V) on the chamber without the flat interface. In this case, the total optical path length is long. An image picture out of focus is obtained, as shown in Fig. 5(c). Then, we gradually increase the voltage on the chamber with the flat interface. The total optical path length gradually becomes shorter. Simultaneously, the image becomes clearer. When the voltage is increased to ~82 V, the image is the clearest, as shown in Fig. 5(d). In the experiment, there is no movement part. The proposed modulator just changes the optical path length to realize refocusing function. From the experiment, we can conclude that the proposed optofluidic modulator can be used in imaging systems to compensate the back focal length effectively. In fact, the proposed optofluidic modulator can also be used in adaptive optics and optical detection system to modulate the optical path.

Compared with the conventional methods, the proposed modulator can obtain a variable optical path length without any movement parts. The optical path is modulated only by a voltage. Therefore, it is very convenient to be integrated into measuring or imaging systems. For example, it can be used in the imaging systems to modulate the optical path to compensate the back focal length. It can also be used in the adaptive optics system to modulate the optical path to compensate the aberrations. And it is possible to create an array out of this structure. However, since one chamber of the device is used only for driving, the aperture opening ratio of each pixel is relatively low. We can reduce the size of the driving chamber to increase the aperture opening ratio, but the tuning range of the optical path will be reduced. Therefore, there is a tradeoff between the aperture opening ratio and the tuning range. In fact, the tuning range of the optical path length can be increased further, because the proposed modulator is based on two liquids, and the optical path tuning range can be further increased by choosing two liquids with larger refractive index difference.

We note that the final prototype has slightly different saturation behavior comparing with that in Fig. 2. It is because the shapes of the chamber are different due to different fabrication methods. For the first prototype as shown in Fig. 2, it is only used for demonstrating the moving behavior. Thus, the material of the sidewall is glass and it is transparent. The shape of the chamber is cubic. However, the material of the second prototype is aluminum as shown in Fig. 3. It is fabricated by precision machining. The shape of the chamber is cylindrical. Thus, the two initial contact angles *θ*_0_ are slightly different due to the shape of chamber. Therefore, the two prototypes of the same design have slightly different saturation behavior.

## Conclusions

In conclusion, we demonstrate an optofluidic variable optical path modulator. The proposed modulator consists of two main chambers. The two chambers are connected by two tubes to form a closed-loop fluidic system. Two immiscible liquids are filled into the two chambers and form two L-L interfaces. A transparent sheet is placed between one L-L interface to get flat interface. When a voltage is applied on the device, the flat interface can move up and down. Thus, we can obtain variable optical path by applying a voltage. To prove the concept, we fabricate an optofluidic device whose largest movable distance is ~7.5 mm and the optical path change is ~1.15 mm. The proposed optofluidic device has potential applications in imaging system, adaptive optics, optical detection and so on.

## Supplementary information

Media 1

Media 2
